# Virus-Associated Lymphomagenesis

**Published:** 2006-06

**Authors:** V. Z. Tarantul

**Affiliations:** *Institute of Molecular Genetics, Russian Academy of Sciences, Moscow, Russia*

**Keywords:** lymphoma, virus, oncogenicity, herpesvirus, epstein-barr virus, hepatitis b virus, HIV, HIV virus HIV, oncogenicity, herpesvirus, epstein-barrvirus, hepatitis cvirus

## Abstract

At least 2 billion people are affected by viral infections worldwide. The infections induce a lot of various human diseases and are one of the main causes of human mortality. In particular, they can lead to development of various human cancers. Up to 15-20% of human cancer incidence can be attributed to viruses. Although viral infections are very common in the general population, only few of them result in clinically relevant lesions. Certain associations between virus infections and malignancy are strong and irrefutable, the others are still speculative. The criteria most often used for determining the causality are the consistence of the association, either epidemiologic or at the molecular level, and oncogenicity of viruses or particular viral genes in animal models or cell cultures. Due to some ambiguity of such a determination, it is instructive to consider by specific cases what evidence is generally accepted as sufficient to establish a causal relation between virus and cancer. Lymphomas are one of the best studied cancer types closely associated with a small but definite range of viruses. Numerous data show a close interrelation between lymphomagenesis and infection by such viruses as Kaposi’s sarcoma herpesvirus (KSHV), Epstein-Barr virus (EBV), hepatitis C virus (HCV), human T-cell leukemia virus (HTLV), and human immunodeficiency virus (HIV). For instance, experiments on monkeys artificially infected with viruses and data on anti-cancer effect of specific antiviral preparations strongly suggest the involvement of viruses in lymphoma development. The present review is devoted to the association of different viruses with human lymphomas and to viral genes potentially involved in the neoplastic process. The recognition of virus involvement in lymphomagenesis may facilitate new strategies for cancer therapy, diagnosis and screening and can lead to a reduction in the number of individuals at risk of disease.

## INTRODUCTION

About a century has passed since Peyton Rous reported that a tumor was transmitted between chickens like an infection disease. At present, increasing evidence indicates that, at least in some cases, viruses are in fact etiologically associated with malignancy and 15-20% of total cancer incidence (1). However, there is still much to be understood in this problem.

When a definite cancer type is not always associated with the presence of a definite virus, the significance of the association of the virus and the cancer is generally uncertain and problematic in terms of etiology. Investigation of molecular mechanisms underlying such associations contributes much to the understanding of virus-cell interaction nature. It has been proved that oncogenes encoded by human tumor viruses play integral roles in the viral conquest of the host cell by subverting crucial and relatively non-redundant regulatory circuits that regulate cellular proliferation, differentiation, apoptosis and life span. Human tumor virus oncoproteins can also disrupt pathways that are necessary for the maintenance of the integrity of the host cell genome. Some viral oncoproteins act as powerful mutator genes and their expression dramatically increases the incidence of host cell mutations with every round of cell division. Others subvert cellular safeguard mechanisms intended to eliminate cells that have acquired abnormalities that interfere with normal cell division. Viruses that encode such activities can contribute to initiation and/or progression of human cancers (2).

All these scenarios of viral effects can be clearly observed in the instance of lymphomagenesis, in which a rather close interconnection between tumor development and viral infection can be most clearly traced. While for most cancers incidence and mortality are decreasing, those of lymphoma, and especially non-Hodgkin’s lymphoma (NHL), are steadily increasing. Research to define reasons for this increase is extensive, but has not yet resolved them. Unlike many other cancer types, smoking appears to be weakly positively associated with the risk of follicular NHL, and alcohol intake is associated with a decreased risk of NHL. The best described risk factor for NHL is immune deficiency; rates of NHL are greatly increased, with relative risks of 10-100 or more, in people with immune deficiency associated with immune suppressive therapy after transplantation, HIV/AIDS, and congenital conditions (3, 4). Another risk factor, a more important one, for some lymphoma subtypes is characteristic of specific infections of some DNA- and RNA-containing human viruses: Epstein-Barr virus (EBV), Kaposi’s Sarcoma Herpesvirus (KSHV), hepatitis C virus (HCV), human T-cell leukemia virus (HTLV), and human immunodeficiency virus (HIV). Each of these viruses can establish a persistent infection in a majority of individuals world-wide or, in some cases, within certain populations where the infection is endemic and the associated lymphomas occur at the highest incidence. However, usually only a minor proportion of the infected hosts will eventually develop lymphoma. In the vast majority of cases, the infection will be either asymptomatic or accompanied by benign proliferation of virus-infected cells. A low number of infected individuals with cancer and a very long latency period suggest that these viruses, although being an important risk factor for lymphoma development in human, are frequently insufficient per se to induce lymphoma. In individual cases, the virus appears to serve as a cofactor of malignancy, and sometimes the association is inconsistent.

This review summarizes basic molecular mechanisms of viral participation in lymphomagenesis and the prospects for future cancer therapy, diagnostic applications, and generation of the most promising avenue for lymphoma prevention.

## DNA-CONTAINING VIRUSES OF PRIMATES

### Epstein-Barr virus

Human gamma-herpesviruses, and in the first place Epstein-Barr virus (EBV), are among the most important viruses that have been for many years discussed in terms of their close association with lymphomagenesis. A hallmark of herpesviruses is their ability to establish a latent infection for the life time of their host. EBV is an omnipresent viral infection and is virtually found in >90% of human population. EBV is also one of the major viruses with human B lymphocyte tropism.

EBV is strongly associated with development of several cancers, in particular with Burkitt’s lymphoma (BL), some subtypes of non-Hodgkin lymphoma (NHL) (nasal NK/T-cell lymphoma, gastric diffuse large B-cell lymphomas), and lymphoproliferative disorders which complicate immune suppression conditions. These EBV-associated neoplasms are characterized by peculiar geographic distributions and distinctive epidemiologic features. Abundant epidemiological, clinical, serological grounds and molecular genetic data have been obtained for a causal relation between the DNA-containing EBV and the formation of endemic (African) Burkitt’s lymphoma (eBL) (see (5)). The link between EBV and eBL proved consistent and became the first of an unexpectedly wide range of associations discovered between virus and tumors. BLs are marked by characteristic translocations between the c-myc gene and immunoglobulin loci (6).

Moreover, there are data in favor of a strong association between EBV and Hodgkin’s disease (HD) (7-10). HD is an unusual lymphoma in which malignant cells, termed Hodgkin/Reed-Sternberg (HRS) cells, comprise less than 1% of the total cell population. In about 50% of classical HD cases, the HRS cells are infected by EBV, suggesting an important role of the virus in HD pathogenesis.

In EBV-positive BL and HD, latent infection of B lymphocytes by the virus is now believed to be an early transforming event (11). Although the infection is latent and does not produce mature viruses, the viral genes are expressed in all tumor cells (5). An important property of EBV that underlies its relation to cancer is its ability to affect B-cell growth regulation and to induce permanent cell transformation. In addition, EBV infection in New World primates leads to lymphomas, and inoculation of peripheral blood mononuclear cells from Epstein-Barr virus-seropositive animals into severe combined immunodeficiency mice results in B-cell lymphoproliferative disorders.

EBV can immortalize human B lymphocytes and controls their resistance to apoptosis. The virus encodes multiple proteins that have profound effects on cellular gene expression. The investigation of the molecular mechanisms of lymphomagenesis in EBV-infected patients has demonstrated that many of the signaling pathways associated with HD are shared among the EBV-encoded proteins, such as EBNA1, EBERs, transforming proteins LMP1 and LMP2, and cellular proteins induced by them. In BL viral expression, they are shared among a more limited number of proteins (mainly EBNA1 and EBERs). LMP1 is essential for EBV transformation of lymphocytes. Transgenic mice expressing the LMP1 enhancer develop lymphoma at a threefold higher incidence than LMP1-negative mice (12). A knockdown of the LMP1 gene by siRNA completely inhibited lymphoma cell proliferation and induced apoptosis (13). LMP1 oncoprotein was shown to induce expression of many important cellular genes that have profound effects on cellular growth. An important role in this process is played by upregulation of constitutive nuclear factor NF-κB, as well as CD30, CD40, tumor necrosis factor TNF-α, and Notch1 interactions (14).

Another viral transforming protein, LMP2, can also induce or suppress and even inactivate expression of cellular genes. In particular, this protein activates Akt that phosphorylates and inactivates GSK3β, causing an increase in β-catenin levels (15), which may contribute to oncogenic properties of EBV-infected B-cells.

EBV may play a role in inhibiting P16(INK4A) expression, thus resulting in a perturbed p16(INK4A)-Rb cell cycle checkpoint (16). It was reported that the dominant effect of viral protein EBNA3C is a decrease in Rb protein levels (17). The loss of suppressor gene products might facilitate lymphomagenesis.

Thus, a number of EBV genes show a strongly manifested oncogenic effect, which was confirmed using a transgenic mice model. It was demonstrated that each of EBNA1, LMP1 or LMP2A genes alone is oncogenic *in vivo*, and all they are important factors contributing to the development of EBV-associated lymphomas (12, 18). In addition, EBV can cause epigenetic modifications that may contribute to viral pathogenesis and tumorigenesis (19). One critical regulatory mechanism utilized by EBV is DNA methylation of cytosine residues in CpG dinucleotides located within promoter regions, which is important for gene silencing and genome integrity (20).

Data obtained with primates are also in favor of EBV involvement in human lymphomagenesis. Several simian rhadinoviruses related to EBV have been found in various Old World primates. Herpes virus saimiri (HVS), a T-lymphotropic agent of squirrel monkeys (Saimiri sciureus), closely related to EBV, infected T-lymphocytes of the natural host (Saimiri sciureus) with no symptoms, but induced acute T-cell lymphomas and other lymphoproliferative diseases in numerous non-natural primate hosts, such as common marmosets or cottontop tamarins (21, 22). An EBV-related simian herpes virus revealed in monkeys and operationally designated as herpes virus Macaca fascicularis (HVMF-I) (23) is also associated with B-cell lymphomagenesis.

The data above suggest that expression of viral genes in most EBV-containing lymphomas is essential for the development of these cancers. Summarizing the available data, it can be concluded that in BL cases EBV is most probably a contributing cofactor. In HD cases EBV might act rather as a factor contributing to lymphoma progression than causing the tumor (24). Endemic patterns of incidence suggest that both genetic and environmental factors contribute to EBV-associated lymphomagenesis.

### Kaposi’s sarcoma-associated herpesvirus

EBV is not the single human gamma-herpesvirus associated with development of lymphoproliferative diseases. Over a decade ago, the first human rhadinovirus (gamma2-herpesvirus), human herpesvirus type 8 (HHV-8) distantly related to EBV, was discovered in Kaposi’s sarcoma (KS) biopsies (25). Since DNA of this virus could be found in all KS forms, the virus was also termed KS-associated herpesvirus (KSHV). This virus was found to be closely associated with rare B-cell lymphoproliferative disorders such as multicentric Castleman’s disease (MCD) and primary effusion lymphoma (PEL), that represent a specific subset of NHLs also known as body cavity-based large-cell lymphoma (26-28).

Since one of the criterions for diagnosis of PEL is KSHV positivity, all PELs are KSHV-positive. Apart from KSHV, PELs contain also other viruses. Most PELs are often coinfected with EBV. KSHV DNA sequences were identified in acquired immunodeficiency syndrome (AIDS-related NHL) patients. Nearly all of AIDS-associated MCDs are associated also with KSHV, whereas only about 50% of non-AIDS-associated MCDs contain KSHV DNA (29). Thus, at least in some cases, the development of KSHV-infected lymphomas can be due to a combined action of this and other viruses (EBV or HIV).

KSHV has about a hundred open-reading frames. Eleven of them are homologs of cellular genes that were hijacked from the host during a long parallel evolution. This virus encodes several viral homologs of cellular cytokines and anti-apoptotic proteins (vIL-6, vBcl-2, vFLIP and others) that can be also involved in cancer development. At least five of KSHV-specific genes display transforming ability both *in vitro* and *in vivo* (30). In particular, the products of kaposin are often regarded as potential lymphomagenesis-stimulating agents. One of kaposin protein isoforms is involved in the regulation of integrin-mediated cell adhesion (31), whereas another one activates the p38/MK2 pathway (32). Lana is one more gene that can be related to lymphoma development. This gene is expressed in all KSHV-associated tumors. The LANA protein is important for the viral genome maintenance and regulates also cellular gene expression. LANA is a transcriptional co-activator of STAT3 (33), it interacts with the tumor suppressor proteins p53 and pRb and promotes chromosomal instability, suggesting that LANA may target these proteins and promote oncogenesis (34-36). The K1 gene, whose expression has been detected in PELs and MCD, is another one KSHV gene probably involved in lymphomagenesis process (37, 38). This gene can signal to induce B-cell activation and transform cells (39, 40). Moreover, K1 activates NFκB and NFAT and induces phosphorylation of several cellular signal transduction proteins, such as Vav, p85, Syk and Akt kinase (41). According to some data, the viral gene vGPCR, homologous to the receptor gene IL-8, can play a definite role in the induction of lymphomagenesis. This gene has been shown to possess a transforming potential and to activate a wide range of cell signaling networks by inducing the transcription of a number of cellular genes involved in cell proliferation and transformation (42, 43). The oncogenic potential of some KSHV genes has been confirmed in experiments with transgenic animals (18, 40).

Thus, a majority of the virus proteins with various properties ensure sufficiently long viral persistence in the human and likely contribute to KSHV-associated lymphomagenesis through the participation in either tumor induction or tumor progression. Since PELs are monoclonal in nature, it can be assumed that KSHV infection precedes tumor progression and may be involved in tumor induction and progression.

An important role of KSHV in pathogenesis of PELs was supported by the data obtained by Fan *et al*. (44), who analyzed gene expression profiles to determine the viral impact on cellular gene expression. It has been shown that KSHV-positive PELs are very different from other lymphomatous effusions and that about 500 differentially expressed genes include apoptosis and cell cycle regulators, transcriptional factors, and signal transduction regulators. Forty genes differentially expressed among KSHV-positive, EBV-positive PEL and KSHV-positive, EBV-negative PEL were identified. The expression of four of these genes (p38δ, GADD45, caspase 1, and SKAP55R), known to be regulators of the mitogen-activated protein kinase pathway, is significantly higher in KSHV-positive, EBV-negative PELs. Based on the data obtained, it can be concluded that the mitogen-activated protein kinase pathway may act as a cofactor for PEL development.

Thus, a strong molecular and epidemiological link between KSHV and PEL suggests that this virus is necessary for development of this malignancy. A causal relation between KSHV and PEL has been also suggested.

It has been shown that anti-herpesvirus drugs that inhibit herpesviral replication, such as ganciclovir, are ineffective against latent viruses (29), and therefore the drugs against tumors that emerge upon KSHV infection are not targeted at the virus itself. For this reason, the search of new drugs specifically directed at individual viral genes presumably involved in lymphoma development is needed.

## RNA-CONTAINING VIRUSES OF PRIMATES

### HCV

Hepatitis C virus (HCV) can be found in more than 200 million people worldwide. Apart from the well documented pathogenic involvement of HCV in hepatic diseases, an increased risk of B cell non-Hodgkin lymphoma has been reported for a minority of HCV infections (45-50). The most frequent types of HCV-associated lymphomas are low-grade B-cell NHL, splenic marginal zone lymphoma, lymphoma of mucosa-associated lymphoid tissue, primary hepatic lymphomas (PHL), and diffuse large cell lymphoma. HCV infection leads to an about 50-fold increased risk of NHLs affecting the liver and major salivary glands and to an about 4-fold increased risk of NHLs in other organs (51-53). Epidemiologic and experimental data strongly suggest an etiopathogenic role of HCV in the development of a subset of NHLs, particularly those complicating the course of some autoimmune diseases, especially of mixed cryoglobulinemia (MC) syndrome (47, 54). A high prevalence (7.5-37%) of chronic HCV has been demonstrated among patients with lymphomas (47, 55, 56). However, the association between HCV infection and B-cell lymphomas is controversial, since it shows a strong regional variation.

Detailed mechanisms responsible for the development of disorders in HCV positive patients still remain unclear. HCV contains an RNA genome, does not contain any obvious oncogenes, and does not integrate into the host genome. According to some data, HCV can induce a mutator phenotype and transform cells by the hit-and-run mechanism (57, 58). A significant role in lymphoma development can be played by HCV-E2, the major envelope protein of HCV. HCV-E2 binds with high affinity CD81, the tetraspanin expressed in several cell types (59). This leads to a preferential activation of naive B lymphocyte proliferation. Such an assumption is supported by the fact that eradication of HCV infection by IFN therapy is associated with normalization of the activation markers expression.

As the frequency of NHL is much lower than that of HCV infection, it is suggested that HCV alone is unable to induce lymphomas, and additional events (cellular or viral) are necessary for obtaining a malignant B-cell phenotype (54). It may be so that only some particular HCV genotypes are involved in lymphomagenesis (46, 60). Alternatively, the course of disease in HCV-positive B-cell NHL patients might be complicated by coinfections with other infectious agents. This possibility has been examined in studies of potential interactions between HCV and other viruses (HIV, EBV or HGV). According to some data (61, 62), there is no evidence for any relation between dual infection by HIV and HCV and increased risk of lymphoma. At present, it is mostly believed that the role of HCV in lymphomagenesis is indirect and related to chronic antigenic stimulation (63).

Nevertheless, encouraging data emerge from recent studies showing that interferon (plus ribavirin) is an attractive therapeutic option for some hepatitis C virus-related low-grade lymphomas (64). Therefore, antiviral therapy is beneficial also in this case.

It cannot be ruled out that some other herpes viruses are involved in lymphomagenesis. For instance, in 247 cases of NHL the overall prevalence of a newly discovered hepatitis G virus RNA positivity was found to be 7.2 % (18/247) (65).

### HTLV/STLV

Some human and simian retroviruses are currently believed to be the etiological agents of primates’ malignant lymphomas. It is the oncogenic action of HTLV/STLV type viruses that has been demonstrated first (66-69). In particular, HTLV-1 is an etiological agent of adult T-cell Leukemia/Lymphoma (ATLL), it is also associated with cutaneous T-cell lymphoma (CTCL). While an estimated 20 million people worldwide are infected with HTLV-1, the infection is endemic in the Caribbean islands, parts of Africa, Southwestern Japan, South America and Italy. After transmission of HTLV-I, 2-5% of carriers are likely to develop ATLL after a long latent period. There are multiple reports where HTLV-1 was found in the peripheral blood or cutaneous lesions of some patients with Mycosis fungoides/Sezary syndrome, but studies did not reveal any evidence for the role of HTLV-1 in this lymphomagenesis (70). HTLV-1 has been also shown to cause T-cell lymphomas in mice (71).

The data obtained with primates are also, though indirectly, in favor of HTLV-1 participation in cancer development. Simian T-lymphotropic virus type 1 (STLV-1) is a C-type retrovirus of nonhuman primates that is genetically and antigenically related to HTLV-1. Similar to HTLV infection in humans, STLV infection has been most often associated with spontaneous malignant lymphomas (leukemia) and lymphoproliferative diseases (66, 67, 72).

However, the mechanism(s) of HTLV-1-mediated T-cell transformation is unclear. Similar to other retroviruses, the HTLV-1 proviral DNA integrates into the human genome but does not produce insertional mutagenesis. To facilitate transmission, HTLV-I increases the number of infected cells through the activity of accessory genes, which are encoded by the pX region (tax, rex, p30, p12, p13, and HTLV-I basic leucine zipper factor (HBZ)). Unlike many animal retroviruses causing cancer, HTLV-1 does not contain any classical oncogenes. However, a non-structural, regulatory and crucial for HTLV-1 replication protein Tax can induce expression of cellular protooncogenes and is necessary and sufficient to transform various cells. Therefore, Tax is considered to be a viral oncoprotein (73, 74). Tax interacts with numerous cellular proteins to reprogram such cellular processes as transcription, cell cycle regulation, DNA repair, and apoptosis (for review see (63, 74, 75)). Moreover, Tax induces persistent activation of NF-kappaB, causing deregulated expression of a large array of cellular genes, which in turn contributes to the induction of T-cell transformation. Through CREB, NF kappaB and SRF pathways Tax transactivates cellular promoters including those of cytokines (IL-13, IL-15), cytokine receptors (IL-2Ralpha) and costimulatory surface receptors (OX40/OX40L). The transactivation results in upregulated protein expression and activated signaling cascades (e.g. Jak/STAT, PI3Kinase, JNK). Tax also stimulates cell growth by direct binding to cyclin-dependent kinase holoenzymes and/or inactivating tumor suppressors (e.g. p53, DLG). Furthermore, Tax silences cellular checkpoints, which guard against DNA structural damage and chromosomal missegregation, thereby favoring manifestation of a mutator phenotype in cells. A number of studies on transgenic mice also demonstrate the lymphomagenic potential of Tax (18). Taken together, these results open a window on the role of Tax in AIDS related oncogenesis. However, Tax expression is often suppressed in ATLL by several mechanisms including genetic changes of the tax gene, deletion/hypermethylation of 5’-LTR. As shown recently, in such cases the HBZ gene might promote proliferation of ATLL cells in the RNA form and play an important role in oncogenesis by HTLV-1 (76).

Thus, according to epidemiological and molecular genetic data, HTLV-1 meets all the criteria of a human tumor virus and can be considered the sole causal agent for ATLL in regions endemic for this cancer. However, a low incidence and a long latency period preceding the occurrence of the disease suggest that additional factors are involved in the development of ATLL. In particular, most malignancies carry aberrations in p16-pRB and/or p53 pathways (77). Thus, somatic mutations of definite cellular genes, most likely leading to disturbance of cell cycle regulation, are required for the development of ATLL.

### HIV/SIV

The incidence of B-cell non-Hodgkin’s lymphoma (NHL) in AIDS patients approximately 200-fold exceeds the normal rate (3, 4), and 5-10% of HIV-infected individuals develop AIDS-related NHL (78). AIDS-NHLs are a heterogeneous group of malignancies. A vast majority of HIV-associated lymphomas are high-grade B-cell lymphomas: Burkitt lymphoma (BL), diffuse large B-cell lymphoma (DLBCL) with centroblastic (CB) features, and DLBCL with immunoblastic (IBL) features (79-86). In HIV-infected individuals, extranodular forms of NHLs are rather frequent (87), e.g. central nervous system lymphoma occurs in 15-25% of cases (88-90). Apart from other lymphomas, cases of HD occurrence upon HIV-infection have been reported, as well as of T-cell lymphomas in 5 10% of cases (83, 91-94). HIV-associated T-cell lymphomas include lymphoblastic lymphoma, PTCL, ALCL and cutaneous T-cell lymphoma.

Coinfection of HIV with other viruses, such as EBV and KSHV, have led to the genesis of previously rare or unrecognized lymphoma subtypes such as plasmablastic lymphoma of the oral cavity and primary effusion lymphomas (PEL). EBV is associated with more than 50% of the lymphomas occurring in the context of HIV infection; KSHV is involved more rarely (62).

The pathological heterogeneity of AIDS-NHLs reflects the heterogeneity of their associated molecular lesions. The number and type of genetic lesions somewhat vary among AIDS-related NHLs according to their histopathologic category and anatomic site of origin. The appearance of AIDS-related NHLs is characterized by the presence of a monoclonal B-cell population that displays a variety of genetic lesions, including, for example, c-myc and bcl-6 genes rearrangement, p53 and ras genes mutations. The expression patterns of Bcl-6 (a protein specific to germinal center (GC) B-cells, and CD138/syndecan-1 (Syn-1), a marker of post-GC B-cell differentiation), showed that the Bcl-6(+)/Syn-1(-) pattern was associated with AIDS-related small and large noncleaved cell lymphomas, while the Bcl-6(-)/Syn-1(+) pattern was associated with AIDS-related large cell immunoblastic lymphoma plasmacytoid and AIDS-related primary effusion lymphomas. The former lymphomas reflect a GC stage of differentiation, whereas the latter derive from B cells that have entered post-GC plasmacell differentiation (95). These findings suggest that more than one pathogenetic mechanism is operational in the development and progression of AIDS-related NHLs.

It has been suggested that tumors arising during HIV-infection are only the result of immunosuppression (96). According to Beral *et al*. (97), the epidemics of AIDS is “a large-scale natural experiment with much to be derived for studying the role of immunodeficiency in cancer formation”. At the same time, there are reasons to believe that lymphomas growing upon HIV infection are conditioned not only by immune deficiency. AIDS related lymphomas are distinct from their counterparts seen in HIV-1 seronegative patients. Firstly, although the tumors occurring upon HIV-infection are generally similar to those associated with immunosuppressive therapy, there are still marked differences between them (for instance, BL is frequently associated with HIV-infection, but it does not appear under the effect of immunosuppressants). Secondly, about 40% of HIV-associated lymphomas were observed in individuals without AIDS symptoms (98). Thirdly, nearly half of all cases of ARL are associated with the presence of a gamma herpesvirus (EBV or KSHV). Fourthly, HIV and its regulatory genes can transform B cell lines and transgenic mice (99-101). And finally, molecular studies have revealed both similarities and differences between HIV-associated and non-HIV-associated lymphomas. Overexpression of some genes in a large proportion of HIV-associated DLBCL as compared to spontaneous DLBCL has been reported (102, 103), although some data contradict this observation (104).

The results obtained for primates were in favor of the association between HIV and lymphoma development. Several authors have reported isolation of different simian immunodeficiency viruses from lymphoma-bearing monkeys (105, 106). For instance, the SIV/Mne virus infectious for both short-tail macaca and macaca rhesus was isolated from the lymph nodes of a short-tail Macaca nemestrina animal that had died of malignant lymphoma (106).

Artificially SIV-infected monkeys also frequently develop different types of lymphoma. This phenomenon was observed, for instance, in 35-40% of SIVsm-infected cynomolgus monkeys Macaca fascicularis (23, 107, 108), and in 13-30% of SIVmac251-infected Macaca mulatta (109, 110). 35% of macaques experimentally infected by SIV/Delta developed EBV related lymphomas (111). Bearing in mind that a considerable amount of artificially SIV-infected monkeys carried lymphomas and taking into consideration the presence of infectious SIV in some tumor cells (108), it can be suggested that SIV is directly involved in the process of lymphomagenesis.

The pathogenicity of HIV in humans and SIV in monkeys appeared to be similar (107, 112, 113), as well as the types of the emerging lymphomas (107, 108, 110, 111, 114). Malignant lymphomas in SIV-infected monkeys were of high or intermediate grade malignancy and, when tested, usually of B-cell origin with morphological features similar to HIV-associated lymphomas, often with extranodal manifestation (111, 114, 115). NHLs of T-cell origin were also reported (108). However, no cases of classical BL or HD development have been hitherto observed.

Like in HIV-infected humans, the presence of EBV was often observed in lymphomas of SIV-infected monkeys (107, 111). For example, a simian homolog to EBV (HVMF-1) was shown in nine cases of lymphomas in 10 cynomolgus monkeys infected with SIV (116). In monkeys, malignant lymphoma can be also induced by the STLV-I (66).

The pathogenic mechanism of HIV-associated lymphomas is still unclear, although there is a hope that the problem of direct involvement of HIV in this process will be solved. HIV proviral DNA integrates into the genomes of infected cells, but insertional mutagenesis is probably in no way responsible for lymphomagenesis. Most likely, a definite role in this process is played by individual viral proteins. Important part in HIV-associated lymphomagenesis is assigned today to the tat gene product (Tat). Extensive evidence indicates that Tat may be a cofactor in the development of AIDS-related neoplasms. The oncogenic potential of Tat has been demonstrated in cell culture and transgenic mice (101, 117). The molecular mechanism underlying Tat’s oncogenic activity may include deregulation of cellular genes. Accordingly, the interaction between Tat and pRb2/p130 seems to result in the deregulation of the control exerted by pRb2/p130 on the cell cycle (118). An oncogenic potential of the tat gene was demonstrated also in transgenic animals (18). Interestingly, Tat can partially repress the processing activity of Dicer which is involved in maturation of cellular microRNAs (119). This uncommon mechanism of the virus-cell interaction might be important for the understanding of the pathological action of HIV.

As to other HIV genes, practically no data suggesting their involvement have been hitherto reported, although it is well known that HIV can epigenetically reprogram genes in infected cells (120), which can also influence lymphoma development. Thus, unlike all the viruses described above, HIV may be presently considered only one of cofactors contributing to lymphoma development.

## CONCLUSIONS

New data are emerging that indicate not only association of individual viruses with definite cancer types but also their causative functions, at least for some of them, in the first place in lymphomas that develop in the course of EBV, KSHV and HTLV-1 infections. Viruses use various scenarios to secure their existence in the cell and to provide for its malignant transformation. They often play a role of epigenetic oncogenesis-causing factors. Increasing evidence shows that viral genes are key players in regulating DNA methylation in cancer cells.

Importantly, there is a profound functional overlap between viral and tumor cell programs (2). A number of pathways altered by these viruses are the same as those accumulating genetic alterations during tumor progression. Viruses often encode proteins that produce growth deregulation in infected cells similar to that due to mutations in tumor cells. Oncogenes encoded by human tumor viruses play integral roles in the viral conquest of the host cell by subverting crucial and relatively non-redundant regulatory circuits that regulate cellular proliferation, differentiation, apoptosis and life span. Some viral oncoproteins act as powerful mutator genes, and their expression dramatically increases the incidence of host cell mutations with every round of cell division. Sometimes, proteins of completely different viruses affect the same cellular metabolic pathways. For example, HTLV-1 and EBV disturb the Notch signaling cascade which affects several key aspects of normal development by regulating differentiation, proliferation and apoptosis. Some genes of KSHV, EBV, HIV and HTLV-1 are associated with malfunctioning of cellular regulatory suppressor genes, such as p53 (36, 74, 121).

At the same time, each virus is also strictly specific. Thus, viral proteins are discriminating biochemical probes that can be used to identify and characterize novel tumor targets. Viruses can be looked upon as small “lamps” that throw light on inner processes concealed in the cell and responsible for malignant transformation of cells.

A recent demonstration that lymphomas associated with viral infection may regress after successful antiviral therapy confirms the role of viruses in lymphomagenesis. In particular, interferon (plus ribavirin) is an attractive therapeutic option for some HCV-related low-grade lymphomas (122). Drugs such as arsenic trioxide combined with IFN-alpha have also been demonstrated to synergize *in vitro* for inducing apoptosis in HTLV-1 infected T cells. These drugs have been used *in vivo* for treating ATLL patients (123). EBV-specific cytotoxic T cells have been used successfully for prophylaxis and treatment of EBV-associated lymphoproliferative diseases (124). The possibility of therapeutic applications for vector-mediated siRNA delivery to control EBV-associated malignant disorders was also shown (125). siRNA-mediated inhibition of EBNA1 expression suppressed the episomal maintenance function of EBNA1 and inhibited tumor cell growth/survival (126). These data suggest that siRNAs against EBNA1 may have therapeutic value in EBV-associated diseases.

Thus, like other carcinogenic agents, viruses undoubtedly favor cancer development. Millions of years of the co-evolution provided enough time for the creation of very complex and intimate interactions between viruses and humans. The programs of viruses aimed at their reproduction in host cells are clearly alien to normal cells and constantly force them. Peaceful co-existence of viruses and their hosts can sometimes persist for a long time but at definite aberrations from the normal state (mutations, metabolism disorders, decreased immunity, senescence etc.) “egoistic” behavior of viruses rather actively forces the cells to malignant transformation.
